# Human Osteology

**Published:** 1886-01

**Authors:** 


HUMAN OSTEOLOGY, COMPRISING A DESCRIPTION OF THE BONES, WITH DELINEATIONS OF THE ATTACHMENTS OF THE MUSCLES, THE GENERAL MICROSCOPIC STRUCTURE OF BONE, AND ITS DEVELOPMENT. *Wood's Library. 1885.*

This work, as the title implies, is devoted to osteology. In many respects the practitioner and the student of medicine will find it a very interesting and practical treatise on this subject. The author has a happy faculty of blending comparative with human anatomy, which enables the student the more readily to fix important points in his memory, by association, and at the same time to enlarge his ideas of anatomy in general.

That portion of the treatise which relates to the microscopic structure of the bones and their development is excellent. The language is consise without being laconic, and the explanations are so clear that every junior student should readily comprehend them; while for the busy practitioner this is an epitome of the knowledge on these subjects, accepted by the experts of the medical profession to-day. The pages given to ossification are particularly good; by way of illustration read, "the fact that bones are developed from several ossific centres, separated by layers of cartilage, is advantageous to growing animals, for it is necessary to have the shaft ossified to support weight, while other parts remain cartilage and diminish concussion, these acting as buffers to break shock, etc." Under microscopy, the discovery of Mr. Queckett is mentioned, in the statement that lacunæ vary in size and shape in the four great classes of animals, whereby the identity of the bone can be established, as to whether it belongs to a mammal, bird, reptile or fish.

The student who reads this part of the work carefully will find himself prepared to comprehend, intelligently, the pathological changes which osseous tissues undergo.

The descriptive portion is most interesting in two particulars; first, in the application of the principles of mechanics to the attachments of the muscles and to the direction of their force; and second, in the constant allusions, by way of comparison, to the analogous structures in the lower animals. F. C. S.

